# Sensory Ion Channel Candidates Inform on the Clinical Course of Pancreatic Cancer and Present Potential Targets for Repurposing of FDA-Approved Agents

**DOI:** 10.3390/jpm12030478

**Published:** 2022-03-16

**Authors:** Wenjie Shi, Chen Li, Thomas Wartmann, Christoph Kahlert, Renfei Du, Aristotelis Perrakis, Thomas Brunner, Roland S. Croner, Ulf D. Kahlert

**Affiliations:** 1 Molecular and Experimental Surgery, University Clinic for General-, Visceral-, Vascular- and Trans-Plantation Surgery, Medical Faculty University Hospital Magdeburg, Otto-von Guericke University, 39120 Magdeburg, Germany; wenjie.shi@uni-oldenburg.de (W.S.); thomas.wartmann@med.ovgu.de (T.W.); aristotelis.perrakis@med.ovgu.de (A.P.); roland.croner@med.ovgu.de (R.S.C.); 2 University Hospital for Gynecology, Pius-Hospital, University Medicine Oldenburg, Carl von Ossietzky University Oldenburg, 26121 Oldenburg, Germany; 3 Department of Biology, Chemistry, Pharmacy, Free University of Berlin, 14195 Berlin, Germany; chen.li@fu-berlin.de; 4 Department of Visceral, Thoracic and Vascular Surgery, University Hospital Carl Gustav Carus, Technische University Dresden, 01307 Dresden, Germany; christoph.kahlert@uniklinikum-dresden.de; 5 Clinic for Neurosurgery, Medical Faculty and University Hospital Düsseldorf, Heinrich-Heine University Düsseldorf, 40225 Düsseldorf, Germany; redu100@uni-duesseldorf.de; 6 Department of Radiation Oncology, Otto-von Guericke University, Leipziger Str. 44, 39120 Magdeburg, Germany; thomas.brunner@med.ovgu.de

**Keywords:** TRP, pancreatic cancer, sensory ion channels, biomarker, TRPC3, TRPC7

## Abstract

Background: Transient receptor potential channels (TRPs) have been demonstrated to take on functions in pancreatic adenocarcinoma (PAAD) biology. However, little data are available that validate the potential of TRP in a clinical translational setting. Methods: A TRPs-related gene signature was constructed based on the Cox regression using a TCGA-PAAD cohort and receiver operating characteristic (ROC) was used to evaluate the predictive ability of this model. Core genes of the signature were screened by a protein-to-protein interaction (PPI) network, and expression validated by two independent datasets. The mutation analysis and gene set enrichment analysis (GSEA) were conducted. Virtual interventions screening was performed to discover substance candidates for the identified target genes. Results: A four TRPs-related gene signature, which contained MCOLN1, PKD1, TRPC3, and TRPC7, was developed and the area under the curve (AUC) was 0.758. Kaplan–Meier analysis revealed that patients with elevated signature score classify as a high-risk group featuring significantly shorter recurrence free survival (RFS) time, compared to the low-risk patients (*p* < 0.001). The gene prediction model also had a good predictive capability for predicting shortened overall survival (OS) and disease-specific survival (DSS) (AUC = 0.680 and AUC = 0.739, respectively). GSEA enrichment revealed the core genes of the signature, TRPC3 and TRPC7, were involved in several cancer-related pathways. TRPC3 mRNA is elevated in cancer tissue compared to control tissue and augmented in tumors with lymph node invasion compared to tumors without signs of lymph node invasion. Virtual substance screening of FDA approved compounds indicates that four small molecular compounds might be potentially selective not only for TRPC3 protein but also as a potential binding partner to TRPC7 protein. Conclusions: Our computational pipeline constructed a four TRP-related gene signature that enables us to predict clinical prognostic value of hitherto unrecognized biomarkers for PAAD. Sensory ion channels TRPC3 and TRPC7 could be the potential therapeutic targets in pancreatic cancer and TRPC3 might be involved in dysregulating mitochondrial functions during PAAD genesis.

## 1. Introduction

As a leading cause of cancer-related death worldwide, the reported overall five-year survival of pancreatic adenocarcinoma (PAAD) under current standard therapy is less than 8% [1,2]. Although surgery and neoadjuvant chemotherapy have improved the prognosis of pancreatic cancer patients, more than 80% of pancreatic cancer patients are diagnosed at an advanced stage with limited long-curative treatment options available [3]. Thus, biomarkers, allowing early and more precise detection, and thereby allowing therapy in premature stages, or supporting the development of new molecular-tailored therapeutics to improve therapy endurance, are highly needed [4,5].

Previous studies have shown that PAAD is a cellular and molecular highly heterogeneous tumor, and both susceptibility gene mutations and environmental factors may lead to the occurrence and devastating progression parameters of the disease [1]. Recently, with the benefit of next-generation genome sequencing (NGS) and bioinformatics technology, rapid advances in molecular stratification of the disease [6], including the classification of predicate expression signatures have sparked hopes for improved combat of this disease in future clinical care [7,8,9].

The transient receptor potential channels (TRPs) are evolutionarily conserved integral membrane proteins classified as a large gene superfamily, containing 28 different genes [10]. TRPs are cationic channels that act as stimulation-induced signal transducers by altering membrane potential or intracellular calcium (Ca^2+^) concentration. The canonical TRP (TRPC) subfamily is most prominently known for containing the founding member of mammalian TRP channels. TRPs, mostly described in to function in neural cells, have been identified to overtake signal sensory function in various cell types [11]. TRPs are discussed to be important mediators of pain and are under current investigation as targets for developing new pain medications [12]. In addition to the role of maintaining tissue homeostasis, some previous reports link malignant disease progression with responses of these channels including for PAAD [13]. However, to our knowledge the relevance of TRP from a translational viewpoint, interrogating NGS data of patient material and clinical course, has been not been assessed.

In the present study, we constructed a TRPs-related gene expression signature to predict the recurrence risk of PAAD patients, and validate its predictability by internal and external validation. Moreover, in a forward-thinking clinical translational attempt, we conducted a gene set enrichment analysis (GSEA) and molecular docking simulations for FDA-approved substances to assume the cellular function of core genes of signature and as a fundament for future therapy-oriented trials in the context of drug repurposing.

## 2. Materials and Methods

### 2.1. Data Obtain and Pre-Processing

**Figure 1:**
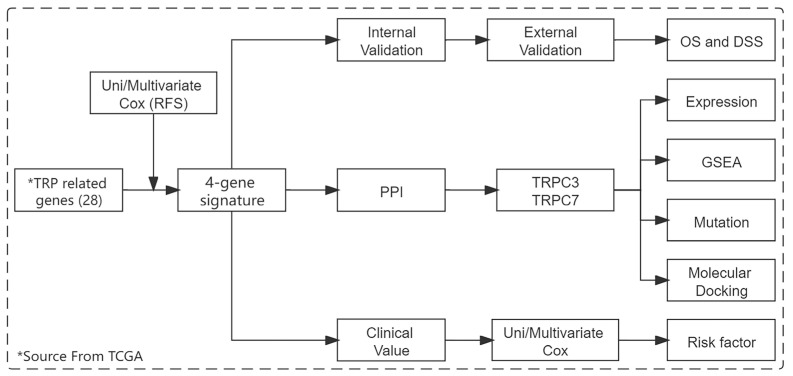
The work flow of this study.

A total of four public datasets were included in this study (TCGA, ICGC, GSE28735, and GSE101448). The TCGA pancreatic adenocarcinoma (PAAD) dataset was used to become the training set for signature construction. The external validation samples were downloaded from the ICGC database. GSE28735 dataset, from the GEO database, which includes 45 cancers and 45 adjacent tissues, was used to validate the core gene expression of signature. GSE101448 dataset was used to validate the core gene expression difference between tumors featuring lymph node invasion vs. tumors without lymph node invasion. According to the clinical information, those patients without a completely recurrence time and status, and recurrence time less than three months are excluded from the cohort. Finally, 136 patients in the TCGA dataset and 111 cases in the ICGA dataset were enrolled in this study. Expression profile data of 28 TRP-related genes were extracted from the gene expression profiles of both datasets and then normalized. The workflow is shown in Figure 1.

### 2.2. Signature Construction

To filter risk genes affecting postoperative recurrence in PAAD patients, we analyzed 28 TRP-related genes by using univariate cox regressions and then input significant candidate genes into a multivariate Cox regression model to identify independent prognosis risk factors. Finally, signature construction was performed based on the coefficients of the multivariate regression results. The model construction equation is as follows. Risk score = coef × A + coef × B + coef × C.

### 2.3. Signature Validation

Randomly assigned extracted fifty percent samples from the training set and all PAAD samples from the ICGC dataset were defined as the internal and external validation set, respectively. In addition, we also defined overall survival (OS) and disease-specific survival (DSS) as new endpoint events, then evaluated the predictive ability of the signature for the new prognosis outcome.

### 2.4. Prognostic Value Evaluation of Signature

Clinical variables are also important for assessing clinical prognosis. To evaluate whether the signature had an independent prognostic value, while compared with clinical characteristics, we combined it with clinical factors and put them into univariate and multivariate Cox regression for future analysis.

### 2.5. Core Gene Screening and Expression Analysis

The protein to protein interaction (PPI) network was used to identify core genes of signature, and the GSE28735 dataset was used to validate the core gene expression between tumor and normal tissues. GSE101448 dataset was used to validate the core gene expression difference between invasion and no invasion tissues.

### 2.6. Core Gene Mutation Analysis

We downloaded the mutation data of the core genes from the TCGA database and divided the samples into high and low groups based on the median expression value of the core gene. Then we analyzed the somatic mutations in the high and low groups of pancreatic patients, respectively, by using the maftools package [14].

### 2.7. GSEA Enrichment Analysis

The functional annotation and pathway enrichment analysis of a single gene can better predict the biological functions that it may be involved in. Here, we performed a GSEA enrichment analysis, including gene oncology (GO) and Kyoto Encyclopedia of Genes and Genomes (KEGG) analysis, on the core genes to clarify the mechanisms in disease. This process is conducted by the R package cluster profile [15].

### 2.8. Virtual Screening

To discover new drug candidates for key proteins of targeting genes, virtual screening was performed. The 3D structures of proteins were downloaded from the Protein Data Bank (PDB) data set (https://www.rcsb.org/ (accessed on 25 January 2022)), and the binding sites of targeting proteins were predicted by DoGSiteScorer from Proteins Plus Server (https://proteins.plus/ (accessed on 25 January 2022)) [16,17]. FDA-approved drugs library as a resource for potential drugs from the ZINC20 database (https://zinc20.docking.org/ (accessed on 25 January 2022)). For this project, the docking was performed and scored using Autodock Vina 1.1.2.

## 3. Results

### 3.1. Risk Gene Identification and Model Construction

The results of univariate Cox regression suggested that eight genes were associated with recurrence in PACA patients, they were MCOLN1, PKD1, TRPC7, TRPV4, MCOLN3, TRPM1, TRPM4, and TRPC3 (Supplementary Table S1). The multivariate cox regression model results demonstrated that four out of those genes, namely, MCOLN1, PKD1, TRPC3, and TRPC7 were independent risk factors associated with the RFS of patients (Table 1). Based on the coefficients of multivariate cox regression, a four-gene signature was constructed, and the model formula was as follows: Riskscore = (−0.048) × MCOLN1 + (−0.042) × PKD1 + 1.072 × TRPC3 + (−2.621) × TRPC7.

### 3.2. Evaluation Model Efficacy

**Figure 2:**
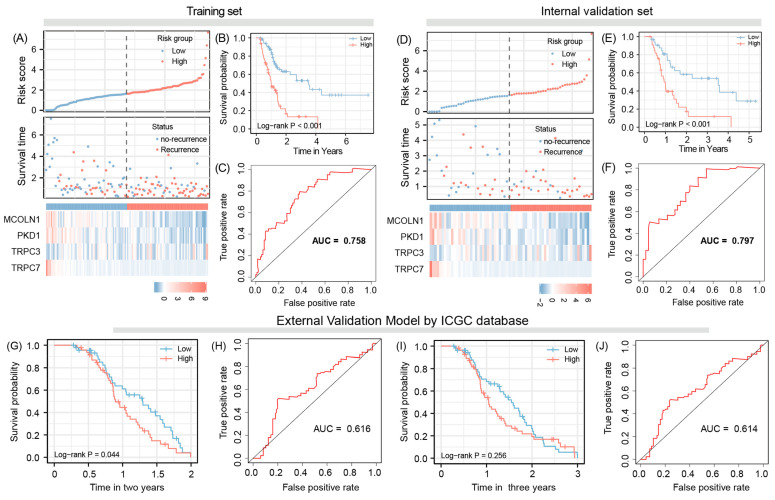
The signature and validation, based on the cox regulation model, a four TRPs-related gene signature was built: risk score—(**A**), survival probability—(**B**), computational performance score of our proposed marker signature evaluated by the area under the receiver operating characteristic curve/ROC (**A**–**C**) and using 50% samples as internal validation (**D**–**F**) and samples from ICGC database as external validation (**G**–**J**).

The area under the curve (AUC) was 0.758, suggesting that the training set signature has good predictive efficacy for RFS. Further Kaplan–Meier analysis revealed that, when patients were divided into high-risk and low-risk groups according to the median value of risk score, patients with the high-risk group had a significantly shorter RFS time, compared to the low-risk patients (*p* < 0.001) (Figure 2A–C).

### 3.3. Model Validation

The internal validation results show that the AUC is 0.797, while two- and three-year external validation results demonstrate that the AUC is 0.616 and 0.614, respectively. In addition, for internal validation samples, patients with the high-risk score also have a short RFS time when compared with patients with low-risk scores (*p* < 0.001). External validation of two years survival data also supports the above conclusion (*p* = 0.044), However, for the external validation of two years data, the comparison between two groups was not statistically significant; although, the separation of RFS curves was indeed very significant (*p* = 0.256) (Figure 2D–J).

### 3.4. Predict Overall Survival and Disease-Specific Survival

**Figure 3:**
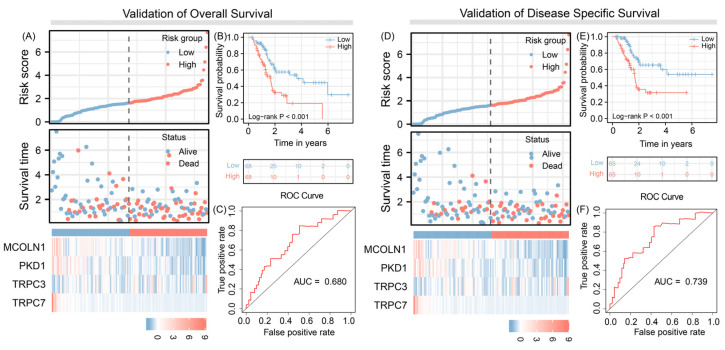
A four TRPs-related gene signature to predict the overall survival: risk score—(**A**), survival probability—(**B**), computational performance score of our proposed marker signature evaluated by the area under the receiver operating characteristic curve/ROC—(**C**) and disease specific survival (similar subdivision of conducted analysis as described for overall survival analysis, (**D**–**F**).

We applied the signature to predict the risk of OS and DSS and the results indicate that the four-gene signature has good predictive efficacy for these outcomes of pancreatic cancer patients (AUC = 0.680 vs. AUC = 0.739, respectively) and patients with high-risk scores have more risk of recurrence, compared to patients with low-risk scores. (Figure 3).

### 3.5. Independent Prognosis Value Evaluation

**Figure 4:**
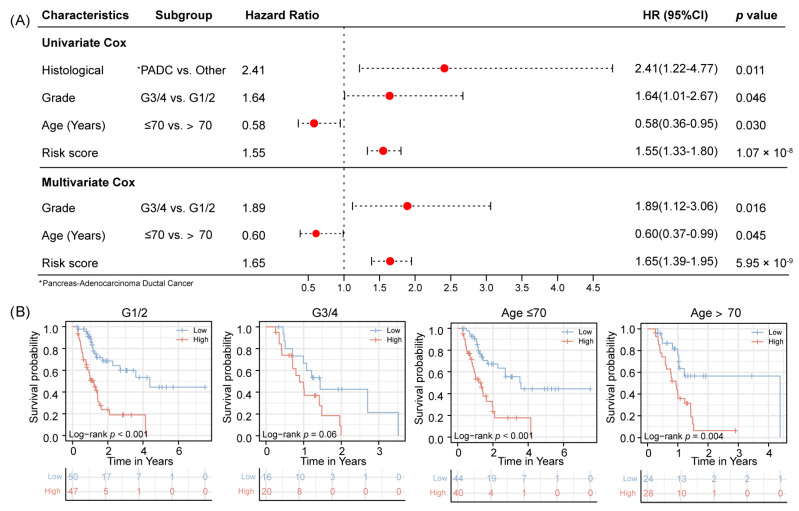
Multivariate regression to discover the risk score was independent risk factors for recurrence (**A**). The subgroup analysis results show that Grade staging and age of the tumor group has a significant negative prognostic value predicting high recurrence risk (**B**).

A four-gene signature, as a variable, was included in the regression model together with clinical variables. The univariate results showed that the histological grade of the tumor, age of patient, and our detected risk scores were independent risk factors for PAAD recurrence. Those results are confirmed by multivariate regression results (Figure 4A). The subgroup analysis results show that G1/2 staging of the tumor group has a significant negative prognostic value predicting high recurrence risk (*p* < 0.001). This conclusion also shows in age subgroups, no matter the patient is older or younger than 70 years old, a low-risk score always means a better prognosis outcome, when compared with the high-risk group (*p* < 0.001 vs. *p* = 0.004). However, when patients with the G3/4 stage, there were no statistical differences between the two groups (*p* = 0.06) (Figure 4B).

### 3.6. Model Core Genes and Expression Validation

**Figure 5:**
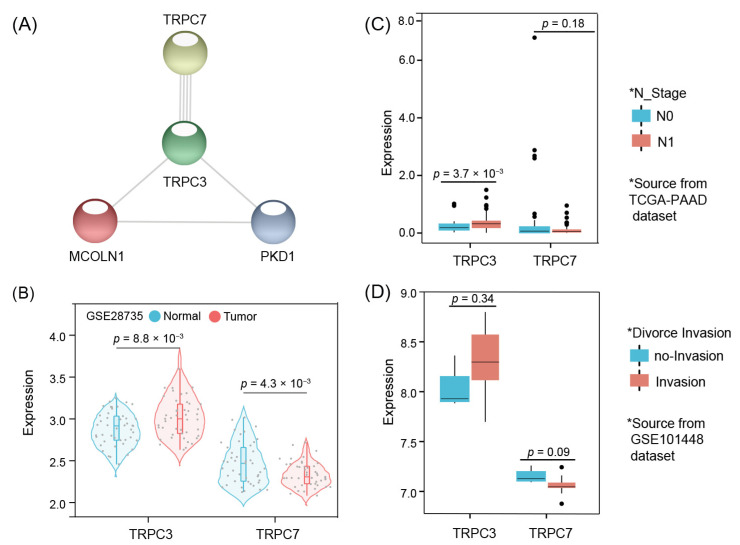
Core genes and expression validation: PPI network show that TRPC3 and TRPC7 were the core genes of the signature (**A**). TRPC3 expression high in tumor tissues, while TRPC7 expression high in normal tissues (**B**), and TRPC3 expression high in lymph positive tissues and invasion tissues, TRPC7 expression high in lymph negative tissues and no-invasion tissues (**C**,**D**).

PPI network analysis results indicate that TRPC3 and TRPC7 are the core genes of the signature (Figure 5A). We firstly used an independent dataset, GSE28735, to verify the expression validation of the genes, and the results suggest that TRPC3 is significantly upregulated in tumor tissues compared to adjacent tissues, with statistically significant differences between groups (*p* = 8.8 × 10^−3^). TRPC7 expression trend is opposite to TRPC3 revealing higher expression value is higher in adjacent samples than in tumor tissues (*p* = 4.3 × 10^−3^) (Figure 5B). Future analysis results show that TRPC3 expression was significantly higher in the tissues of patients with lymph node metastases when compared to those with lymph node-negative (*p* = 3.7 × 10^−3^) (Figure 5C). The expression of TRPC7 was lower in tissues that invasion when compared with samples without invasion; although, it shows no significance between the two groups (*p* = 0.09) (Figure 5D).

### 3.7. Mutation Results

**Figure 6:**
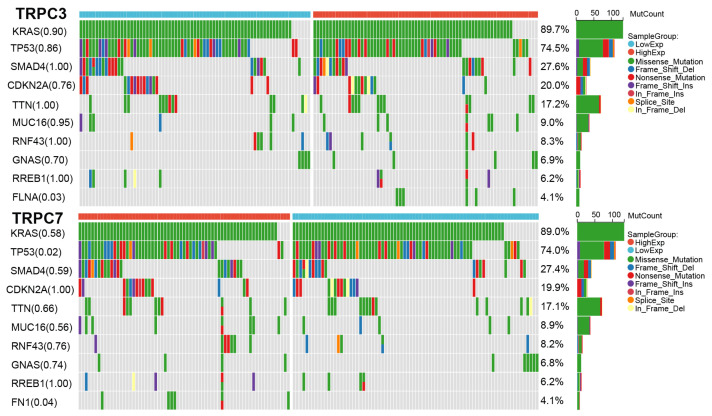
The somatic mutations of high and low groups according to median expression value of TRPC3 and TRPC7 genes.

The waterfall chart of mutation analysis shows that in tumor samples positive for TRPC3 expression, they are also often highly mutated in KRAS gene loci (89.7%), and that the specific mutation type is missense mutation. This conclusion is also applicable for samples with positive TRPC7 mRNA abundancy (89.0%), meaning that in any case of positive signal for TRPC3 or TRPC7 expression in a given tumor, the tumor cells frequently possesses the prominent mTOR pathway activating mutation. In addition, TP53 mutation is also frequent in TRPC3/TRPC7 positive samples (74.5% vs. 74%, respectively). In the high expression group of TRPC3 and lower expression group of TRPC7, missense mutations are also the main mutation type of TP53. Of note, the difference in TP53 mutation frequency between high- and low-expressing tumors of TRPC3 was not observed; however, this difference was very significant between the high and low groups of TRPC7 (*p* = 0.86 vs. *p* = 0.02, respectively) further calling for investigation of the biological function of TRPC7 in the context of an existing known prominent hallmark tumor mutation in TP53 gene. (Figure 6).

### 3.8. Functional Annotation

GO functional enrichment analysis suggested that the TRPC3 gene is associated with mitochondrial translational termination and elongation, and regulation of the humoral immune response. TRPC7 gene enrichment analysis results demonstrate that this gene could be enriched due to the dysregulation of dephosphorylation, protein dephosphorylation, and positive regulation of cytokine production (Supplementary Table S2).

### 3.9. Pathway Enrichment Analysis

Single-gene GSEA results show that up-regulated TRPC3 gene is associated with activation of the ECM-receptor interaction networks as well as stem cell pathway Hippo signalling. The down-regulated TRPC7 gene may activate the Ras signaling pathway and seems to be associated to dysregulations of cellular metabolic pathways (Supplementary Table S3).

### 3.10. Candidate Compound Interventions

**Figure 7:**
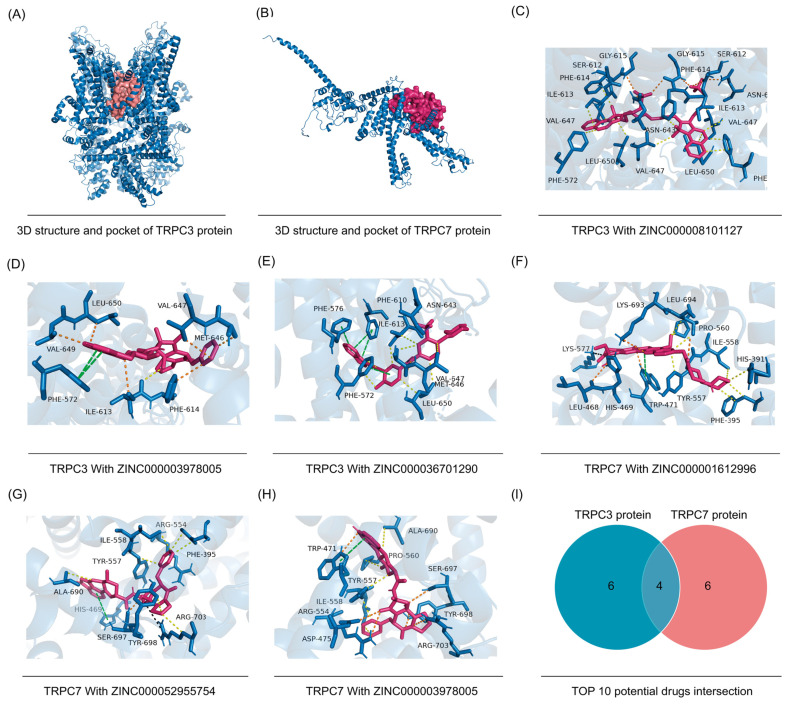
Virtual screening potential drugs for core protein: The 3D structures of TRPC3 and TRPC7 protein (**A**,**B**); the Top 3 docking structure of hub proteins with small molecular drugs(**C**–**H**). (I) Top 10 drugs intersection.

Three-dimensional structures of TRPC3 and TRPC7 are shown in Figure 7A,B. The Top 10 substance drugs, ranked by affinity, are shown in Table 2, and the Top 3 docking structure of hub proteins and small molecular drugs are shown in Figure 7C–H. Future analysis results show that in TOP 10 highest bind score drugs, four small molecular compounds, ZINC000001612996, ZINC000052955754, ZINC000003978005, and ZINC000006716957 are not only potential binding for TRPC3 but also as a potential interacting agent of TRPC7 (Figure 7I).

## 4. Discussion

TRPs are cationic channels that act as stimulation-induced signal transducers by altering the membrane potential or intracellular calcium (Ca^2+^) concentration. TRPC subfamily is most prominently known for containing the founding member of mammalian TRP channels [18,19,20]. TRP expression is widely distributed in neuronal tissues, but not limited to them, as reports on their role on epithelial and endothelial cells have been published [21,22]. They are described as acting as sensors for various environmental stresses including mechanostresses such as tissue injury, changes in temperature, pH value or osmolality, as well as volatile chemicals, cytokines, and plant compounds [23]. Given this pan-tissue relevance, and the emergence of selected TRP genes as molecular surgery targets and tumor-agnostic targets, we sought to investigate the relevance of the TRP class in molecular data on clinical cancer specimens focusing on PAAD. To do so, we interrogated the globally considered forefront genome datasets and applied various state-of-the-art computational methods. Our results enabled us to construct a TRPs-related gene signature to predict the recurrence risk of PAAD patients, classify selected candidates of this gene signature’s possible therapeutic targets, and potentially predict the candidate drugs for core genes of this signature.

The results of our work are novel in several aspects. On the one hand, this is the first evidence showing TRP member genes to possess clinical predictive value, tested on clinical samples of PAAD using expression sequencing. So far, TRP biology in PAAD was assessed to some extent in preclinical models mostly. On the other hand, we show that elevated expression of TRPC3 is associated with shortened RFS, OS, and DSS, is upregulated in N1 compared to N0 tumor cases, and tends to be enriched in the invasive tumor area; our data indicates TRCP3 may possess pro-tumorigenic potential in PAAD. To propose a possible downstream mode of action, we want to point out the comparative results on gene enrichment analysis in TRPC3-high and TRPC3-low cases. We identify a positive correlation between elevated TRPC3 expression and Hippo stem cell pathway activation. Hippo activation is emerging as a therapeutic target for treating PAAD as it is believed to promote the maintenance of tumor stem cells [24]. Interestingly, from physiology-based research, TRPC3 was identified as a prerequisite for the pluripotency and differentiation potential of murine embryonic stem cells [25,26]. Regarding TRPC7, an interesting very recent study by Hsu and colleagues found that TRPC7 acts as a mechanosensitive receptor in the skin and transmits stress signal of ultraviolet B (UVB) to initiate skin aging by augmentation of the production of cellular reactive oxygen [27]. Little is known if TRPC7 plays a role in stem cell biology. In the context of cardiomyocyte research, it was found that low levels of TRPC are associated with the pluripotency stage of the cells TRPC7 [28]. Yang et al. found that TRPC7 expression is increased in epithelial differentiated cells during tooth sperm development [29]. In addition, as we see the correlation of reduced expression of TRPC7 and shortened RFS and a trend to be reduced in the invasive tumor area, it let us speculate that TRPC7 may have tumor-suppressive roles or be an activator regulatory network that diminishes the tumor. In support, GSEA found a prominent cancer hallmark signaling network of RAS signaling to be amongst the most inversely correlated to activation to TRPC7 expression. Strikingly, we propose possible downstream pathways by our result presentation. In our TRPC3 analysis, three out of the top four significant regulated gene ontology networks reveal molecular signal transduction cascades controlling mitochondrial functions, namely mitochondrial translational termination mitochondrial translational elongation and ATP synthesis coupled electron transport (Supplementary Table S2), are dysregulated. TRPC3 over-activation might manifest in the disturbance of the cellular metabolism in pancreatic tumors, but a functional test with genetically engineered PAAD cells to establish isogenic TRPC3 activation conditions similar as performed previously are needed to test this hypothesis [30].

Needless to say, our introduced speculative-driven assumptions need mechanistic validation in an experimental controlled setting to prove our hypothesis. Nevertheless, our data taken together provide evidence that the relevance of TRPC3 over-activation as a disease-agnostic therapeutic target expands to PAAD [31]. To our knowledge our report is the first to introduce TRPC7 in the context of pancreas biology or pancreas carcinogenesis. Applying TRPC7 activation strategies, similar to what has been established for TRPV1 as a molecular surgery target, might help to establish niche factors that combat PAAD cell survival. Our in silico substance screen brought up several intriguing results. First, TRPC3 seems to be able to interact with indocyanine green (ICG), a clinically established dye for intraoperative fluorescence discrimination of target tissue during hepatocellular surgery. Recent clinical reports promote the use of ICG to detect pancreatic cancer lesions [32,33]. Our prediction data suggests a novel possible contributing factor for a mechanistic explanation to establish fluorescence-guided pancreatic surgery. Moreover, in the top suggested candidate genes, for both core genes, we find two prominent anti-migraine drugs, ergotamine, and dihydroergotamine. Ergotamine is reported to have side effects on neurotransmitter networks and might also act on TRPC activation. Ergotamine is well tolerated in patients, offering interesting translational perspectives for clinical feasibly of potential repurpose as cancer therapy. To the best of our knowledge, no such repurposing has been studied in a PAAD cancer model system. Previously, a chemical reduced version of ergotamine, dihydroergotamine, was found to induce apoptosis and mitophagy in the context of lung cancer by disrupting mitochondrial functionality [34].

Despite undertaking enormous efforts, clinicians and drug makers are due to establish treatment options with long-lasting curative effects to patients suffering from advanced pancreatic cancer [35,36]. Advances in technologies in molecular and cell biology have enabled the scientific stakeholders to come up with novel targets that may overcome this long-lasting dilemma. Our study confirmed that ZINC000001612996, ZINC000052955754, ZINC000003978005, and ZINC000006716957 might be small molecule drugs acting as co-ligands of TRPC3 and TRPC7 by the molecular docking technique, and this result suggests that these four small molecule drugs have potential as targeted therapeutic agents for pancreatic cancer. ZINC000001612996, also known as irinotecan, has been shown to significantly prolong patient OS for the treatment of metastatic pancreatic cancer [37]. ZINC000006716957 (nilotinib), as a tyrosine kinase inhibitor, is currently used primarily for the treatment of chronic myeloid leukemia [38]. Although the drug itself has not been reported in pancreatic cancer, other members of tyrosine kinase inhibitors, such as erlotinib, ruxolitinib, and trastuzumab, have demonstrated great superiority in targeted therapy for pancreatic cancer [39,40,41]. This result indicates that nilotinib maybe also has the potential valuable in the treatment of pancreatic cancer targeting. Furthermore, considering that there is a co-activation mechanism of TRPC3 and TRPC7 protein [42] and the expression of these two genes is negatively correlated in tumor tissues, we guess that the overlapping small molecule anti-tumor drugs are likely to be agonists of TRPC7 protein and inhibitors of TRPC3 protein. However, more evidence needs to be provided by follow-up experiments on functional level. Moreover, a dedicated cell-type specific analysis of TRPC3/TRPC7 mRNA abundancy in different tumor types that constitute PAAD parenchyma, such as peripheral nerve cells, immune cells, or vasculature compared to tumor cells, would be very helpful to narrow down the potential mechanism underlying the clinical prognostic value of our discovered biomarkers. Additionally, investigating whether an association of episodes of pain symptoms of the cancer patients and their relative TRPC3/TRPC7 activation in the tumor tissue exists is desirable. The results would surely be insightful to validate if the identified molecules are associated with dysregulated sensory symptoms in patients under disease burden. At this point, we are not in possession of such a dataset, but we have initiated project activities to commence a prospective clinical in our center trail to obtain one. Since we are strongly convinced about the diagnostic biomarker potential of TRPC3 and TRPC7 transcript abundancy in tumor specimens, targeted attempts via rapid-to-apply technologies such as RT-qPCR or target-amplification free CRISPR/Cas diagnostics instead of OMICS-acquisition would enable better applicability/dissemination potential of our markers. Both assays for targeted mRNA expression test could be conducted either on surgical resection of tumor or biopsies—both samples are retrieved during standard surgical oncology care—with moderate infrastructure requirements. Such biomarker-based stratification of tumors based on tumor tissue analysis may help to prospectively inform on the need and selection optimal type of adjuvant/neoadjuvant therapy. On the other hand, ideally clinical prediction would be achieved by analyzing biomarkers on patient material that is acquired by minimal invasive interventions such in as body fluids. At this point we do not know if informing blood signatures, either certain characteristics of routinely assessed lab values or molecular traits, inform on TRPC-activation status in the tumor. To test this shall be the focus of future projects aiming to enlarge our future portfolio of PAAD-directed point of care testing options.

## 5. Conclusions

The transcriptional activation of members of sensory ion channels in PAAD tissue possess predictive value for the clinical course of patients suffering from the disease and may help to improve stratification of tumors according to invasive potential. Functional validation to test predicted drug repurposes and for validation of the prognostic value in prospective trials are needed to prove our hypothesis.

## Figures and Tables

**Table 1:** Four prognostic genes significantly associated with RFS.

Gene Symbol	Coef	Hazard Ratio	95%CI (Low)	95%CI (High)	*p* Value
MCOLN1	−0.048	0.953	0.913	0.995	0.030
PKD1	−0.042	0.959	0.910	1.010	0.115
TRPC3	1.072	2.922	1.230	6.940	0.015
TRPC7	−2.622	0.073	0.009	0.619	0.016

**Table 2:** Top 10 potential binding substances to TRPC3 and TRPC7 protein.

Protein Name	Affinity	ZINC_ID	Drug Name
TRPC3	−10.9	ZINC000008101127	Indocyanine Green
TRPC3	−10.7	ZINC000003978005	Dihydroergotamine
TRPC3	−10.6	ZINC000036701290	Ponatinib
TRPC3	−10.4	ZINC000000896717	Accolate
TRPC3	−10.2	ZINC000164760756	Olysio
TRPC3	−10.2	ZINC000052955754	Ergotamine
TRPC3	−10.2	ZINC000006716957	Nilotinib
TRPC3	−10.1	ZINC000068204830	Daclatasvir
TRPC3	−10.0	ZINC000001612996	Irinotecan
TRPC3	−10.0	ZINC000026664090	Sqv
TRPC7	−12.2	ZINC000001612996	Irinotecan
TRPC7	−11.8	ZINC000052955754	Ergotamine
TRPC7	−11.8	ZINC000003978005	Dihydroergotamine
TRPC7	−11.7	ZINC000006716957	Nilotinib
TRPC7	−11.6	ZINC000066166864	Alectinib
TRPC7	−11.6	ZINC000084668739	Lifitegrast
TRPC7	−11.4	ZINC000064033452	Lumacaftor
TRPC7	−11.3	ZINC000004214700	Paliperidone
TRPC7	−11.2	ZINC000000538312	Risperdal
TRPC7	−11.2	ZINC000003932831	Avodart

## Data Availability

All of these data can be obtained from the public databases TCGA and GEO.

## Supplementary Materials

The following are available online at https://www.mdpi.com/article/10.3390/jpm12030478/s1, Table S1: Using batch cox regression to analysis TRPs-related genes with prognosis factors, Table S2: Gene Oncology enrichment of TRPC3 and TRPC7 respectively, Table S3: KEGG pathway enrichment of TRPC3 and TRPC7 respectively.
