# Supplementary material for: Sensory Ion Channel Candidates Inform on the Clinical Course of Pancreatic Cancer and Present Potential Targets for Repurposing of FDA-Approved Agents
Source: J Pers Med. 2022 Mar 16;12(3):478. doi: 10.3390/jpm12030478 (PMC8950951; doi:10.3390/jpm12030478)
Supplement: Supplementary file 1 [file jpm-12-00478-s001.zip › Supplement Table S2.pdf]

Table S2. Gene Oncology enrichment of TRPC3 and TRPC7 respectively

**TRPC3**				
**ID**	**Description**	**EnrichmentScore**	**NES**	**pvalue**
GO:0050911	detection of chemical stimulus involved in sensory perception of smell	-0.71	-3.95	1.45E-10
GO:0070126	mitochondrial translational termination	-0.52	-3.74	9.51E-09
GO:0070125	mitochondrial translational elongation	-0.52	-3.74	5.19E-09
GO:0032543	mitochondrial translation	-0.44	-3.36	1.00E-10
GO:0042773	ATP synthesis coupled electron transport	-0.45	-3.25	7.21E-07
GO:0099024	plasma membrane invagination	0.74	2.55	1.00E-10
GO:0002920	regulation of humoral immune response	0.78	2.66	1.00E-10
GO:0030449	regulation of complement activation	0.79	2.69	1.00E-10
GO:0006956	complement activation	0.78	2.71	1.00E-10
GO:0002455	humoral immune response mediated by circulating immunoglobulin	0.79	2.75	1.00E-10

**TRPC7**				
**ID**	**Description**	**EnrichmentScore**	**NES**	**pvalue**
GO:0035303	regulation of dephosphorylation	0.59	2.01	3.07E-03
GO:0016050	vesicle organization	0.59	2.29	1.44E-04
GO:0051656	establishment of organelle localization	0.59	2.28	1.49E-04
GO:0007018	microtubule-based movement	0.58	2.24	1.75E-04
GO:0006470	protein dephosphorylation	0.58	1.97	4.04E-03
GO:0001819	positive regulation of cytokine production	-0.56	-2.26	8.58E-04
GO:0031424	keratinization	-0.57	-3.05	2.13E-07
GO:0022617	extracellular matrix disassembly	-0.62	-2.31	9.32E-04
GO:0070268	cornification	-0.62	-3.22	2.46E-08
GO:0018149	peptide cross-linking	-0.72	-2.57	5.72E-05
