# Supplementary material for: Sensory Ion Channel Candidates Inform on the Clinical Course of Pancreatic Cancer and Present Potential Targets for Repurposing of FDA-Approved Agents
Source: J Pers Med. 2022 Mar 16;12(3):478. doi: 10.3390/jpm12030478 (PMC8950951; doi:10.3390/jpm12030478)
Supplement: Supplementary file 1 [file jpm-12-00478-s001.zip › Supplement Table S3.pdf]

Table S3. KEGG pathway enrichment of TRPC3 and TRPC7 respectively

**TRPC3**				
**ID**	**Description**	**EnrichmentScore**	**NES**	**pvalue**
hsa05150	Staphylococcus aureus infection	0.70	2.27	1.00E-10
hsa05144	Malaria	0.70	2.21	1.11E-09
hsa04610	Complement and coagulation cascades	0.70	2.22	2.29E-10
hsa04974	Protein digestion and absorption	0.69	2.24	1.00E-10
hsa00532	Glycosaminoglycan biosynthesis	0.67	1.87	2.60E-04
hsa05322	Systemic lupus erythematosus	0.67	2.08	1.63E-07
hsa05310	Asthma	0.67	1.95	5.98E-05
hsa04640	Hematopoietic cell lineage	0.65	2.17	1.00E-10
hsa04512	ECM-receptor interaction	0.63	2.08	1.83E-10
hsa04392	Hippo signaling pathway	0.62	1.81	4.49E-04
**TRPC7**				
**ID**	**Description**	**EnrichmentScore**	**NES**	**pvalue**
hsa04972	Pancreatic secretion	-0.77	-4.45	1.00E-10
hsa04970	Salivary secretion	-0.73	-2.64	3.00E-05
hsa04975	Fat digestion and absorption	-0.67	-2.52	9.79E-05
hsa00500	Starch and sucrose metabolism	-0.57	-2.20	0.001
hsa04014	Ras signaling pathway	-0.55	-2.05	0.004
hsa04974	Protein digestion and absorption	-0.55	-2.97	2.18E-06
hsa04973	Carbohydrate digestion and absorption	-0.53	-2.17	0.001
hsa04060	Cytokine-cytokine receptor interaction	-0.52	-1.94	0.007
hsa05200	Pathways in cancer	-0.39	-1.90	0.005
hsa01100	Metabolic pathways	-0.24	-1.85	0.004
